# *Salmonella* Typhimurium undergoes distinct genetic adaption during chronic infections of mice

**DOI:** 10.1186/s12866-016-0646-2

**Published:** 2016-03-08

**Authors:** Emilie Søndberg, Lotte Jelsbak

**Affiliations:** Department of Biology, Copenhagen University, Ole Maaløes Vej 5, DK-2200 Copenhagen N, Denmark; Department of Science, Systems and Models, Roskilde University, Universitetsvej 1, DK-4000 Roskilde, Denmark

**Keywords:** Patho-adaptation, Host-interaction, Metabolism, Virulence, Transmission

## Abstract

**Background:**

Typhoid fever caused by *Salmonella enterica* serovar Typhi (*S*. Typhi) is a severe systemic human disease and endemic in regions of the world with poor drinking water quality and sewage treatment facilities. A significant number of patients become asymptomatic life-long carriers of *S*. Typhi and serve as the reservoir for the disease. The specific mechanisms and adaptive strategies enabling *S*. Typhi to survive inside the host for extended periods are incompletely understood. Yet, elucidation of these processes is of major importance for improvement of therapeutic strategies.

In the current study genetic adaptation during experimental chronic *S*. Typhimurium infections of mice, an established model of chronic typhoid fever, was probed as an approach for studying the molecular mechanisms of host-adaptation during long-term host-association.

**Results:**

Individually sequence-tagged wild type strains of *S.* Typhimurium 4/74 were used to establish chronic infections of 129X1/SvJ mice. Over the course of infections, *S*. Typhimurium bacteria were isolated from feces and from livers and spleens upon termination of the experiment. In all samples dominant clones were identified and select clones were subjected to whole genome sequencing. Dominant clones isolated from either systemic organs or fecal samples exhibited distinct single nucleotide polymorphisms (SNPs). One mouse appeared to have distinct adapted clones in the spleen and liver, respectively. Three mice were colonized in the intestines by the same clone containing the same non-synonymous SNP in a transcriptional regulator, *kdgR*, of metabolic genes. This likely indicates transmission of this clone between mice. The mutation was tracked to have occurred prior to 2 weeks post infection in one of the three mice and had subsequently been transmitted to the other two mice. Re-infection with this clone confirmed that it is superior to the wild type for intestinal colonization.

**Conclusions:**

During 4 to 6 weeks of chronic infections, *S*. Typhimurium acquired distinct SNPs in known regulators of metabolic and virulence genes. One SNP, the *kdgR*-SNP was confirmed to confer selective advantage during chronic infections and constitute a true patho-adaptive mutation. Together, the results provide evidence for rapid genetic adaptation to the host of *S*. Typhimurium and validate experimental evolution in the context of host infection as a strategy for elucidating pathogen host interactions at the molecular level.

**Electronic supplementary material:**

The online version of this article (doi:10.1186/s12866-016-0646-2) contains supplementary material, which is available to authorized users.

## Background

*Salmonella enterica* are Gram-negative intracellular pathogens able to cause a variety of infections in both human and animals [1]. The species *S. enterica* constitutes >2500 different serovars, and where serovars with a broad host range like *Salmonella enterica* serovar Typhimurium (*S*. Typhimurium) mainly cause relatively mild gastrointestinal infections, host restricted serovars like *S.* Typhi (human restricted), typically cause severe systemic disease (typhoid fever) of their hosts. Furthermore, about 1–6 % of human typhoid patients become asymptomatic chronic carriers [2] and sporadically excrete bacteria in their stool thus serving as the reservoir for the disease. *S*. Typhimurium causes a typhoid-like systemic infection in mice and functions as a model for typhoid fever. Depending on the mouse strain it is possible to study both acute and chronic *S*. Typhimurium infections. Specifically, a mouse strain containing the wild type allele of the Slc11a1 gene serves as a robust model for studying asymptomatic chronic *Salmonella* infections [3]. During systemic chronic infections of these mice, *S*. Typhimurium reside in macrophages of the spleen, liver and mesenteric lymph nodes for up to 1 year with sporadic excretion of *S*. Typhimurium bacteria in the feces [3]. A negative genetic screen has shown that virulence factors associated with systemic acute infections, including the ones encoded by Salmonella Pathogenicity Island 2 (SPI2) [4], are also essential for establishing chronic infections [5]. However, the specific contributions of individual factors and the metabolic requirements during long-term host association are not fully understood.

In a few natural chronic bacterial infections of humans, the temporal occurrence of adaptive mutations over the course of months to years of infection has recently been delineated [6, 7]. For example, during chronic lung infections of Cystic Fibrosis patients, it has been documented how *Pseudomonas aeruginosa* acquire adaptive mutations facilitating persistence and survival in the host over the course infection [8]. The adapted clones retain the ability to transmit to and infect new patients and represent examples of bacterial evolution and host adaptation studied in real time. These studies have provided new insight into specific adaptive strategies favoring successful chronic infections by revealing the molecular signatures of pathogen host interactions. Similar studies exploring the level of within host adaptive evolution of *S*. Typhi during chronic infections are lacking, partly due to difficulties in identifying chronic carriers and maintaining an extended temporal sampling protocol, as these patients are often asymptomatic. In the present paper, using a previously established tagged strain approach [9], the level of genetic adaptation of *S*. Typhimurium during experimental chronic infections of mice was investigated. To this end, dominant clones isolated 4 to 6 weeks post infection were subjected to whole genome sequencing to identify potential adaptive mutations. In all mice, dominant *S*. Typhimurium clones isolated from either systemic organs or the fecal samples exhibited distinct single nucleotide polymorphisms (SNPs). Three mice were colonized in the intestines by the same clone containing the same non-synonymous SNP in a transcriptional regulator (*kdgR*) of metabolic genes, indicating transmission of this clone between mice. Further investigations confirmed that this clone was superior to the wild type for intestinal colonization presumably due to changes in the regulatory activity of the affected protein.

Together, these results provide evidence for rapid genetic adaptation to the host of *S*. Typhimurium and validate experimental evolution in the context of host infection as a strategy for elucidating pathogen host interactions at the molecular level.

## Results and discussion

### Dominant Salmonella clones emerge during chronic infections

Genetic adaptation of *S*. Typhimurium was investigated during experimental 6 week chronic infections of mice. To identify dominant *S*. Typhimurium clones emerging over the course of the infection and to be able to follow the dynamics of individual clones within the same mouse over time, a previously established tagged strain approach was employed [9]. 8 isogenic strains were constructed, each with a unique 40-bp sequence identifier inserted into the *malX*-*malY* pseudogene region of the wild-type *S*. Typhimurium strain ST4/74. These strains are termed wild-type isogenic tagged strains, or WITS. The WITS are phenotypically identical, but can be distinguished based on the unique sequence enabling investigations into infection dynamics and identification of dominant clones. A pool, with equal distributions of the 8 WITS, was used to infect five female 129X1/SvJ mice via the intraperitoneal (IP) route of infection with an infectious dose of 10^4^ bacteria. This mouse strain contains the Slc11a1 gene (*Nramp1*) which encodes a divalent cation transporter*.* The wild type allele of this gene confers partial resistance to *Salmonella* infection primarily due to divalent cation starvation of intracellular *S*. Typhimurium [10]. It is a robust model for studying chronic *Salmonella* infections with none or only modest symptoms of infection at relatively low infective doses [3] and consequently a very convenient model for studying long-term pathogen-host interactions. Feces were collected over the course of the infection from individual mice and the presence of *S.* Typhimurium was determined by exploiting the kanamycin resistance introduced together with the WITS during strain construction. Two mice had only few or no *S*. Typhimurium bacteria in their feces prior to 4 weeks of infection (Table 1). Whereas mouse 5B, 5D, and 5E  had 10^4^ cfu of *S*. Typhimurium per gram feces after 2 weeks. It is important to note that mice were not pre-treated with streptomycin, so any *S*. Typhimurium clones colonizing the intestines are competing for this niche with the resident microbiota of the mice. Furthermore, as mice were inoculated IP, *S*. Typhimurium isolated from the feces have been circulating in the macrophages prior to reaching the intestines from the liver, gall-bladder or mesenteric lymph-nodes [11]. Two mice (5B and 5D) had to be euthanized after 4 weeks due to symptoms of malaise. After 6 weeks of infection, remaining mice were euthanized and *S*. Typhimurium bacteria in the spleens and livers of all mice were enumerated (Table 1).

**Table 1:** *S*. Typhimurium CFU of samples following i.p. infection

Mouse ID	Per gram feces, 2 weeks post infection	Per gram feces, 4 weeks post infection	Per gram feces, 6 weeks post infection	Per spleen	Per liver
5A	<10^2^	4 × 10^5^	1.2 × 10^4^	3.3 × 10^4^	6.6 × 10^3^
5B	6.5 × 10^4^	1.6 × 10^4^	nd	1.4 × 10^4^	4.6 × 10^4^
5C	<10^2^	3.7 × 10^4^	5 × 10^6^	1.5 × 10^4^	2 × 10^3^
5D	1 × 10^4^	1.4 × 10^4^	nd	2.2 × 10^5^	5.4 × 10^4^
5E	7.5 × 10^3^	nd	6 × 10^6^	7.6 × 10^4^	6.8 × 10^5^

**Fig. 1:**
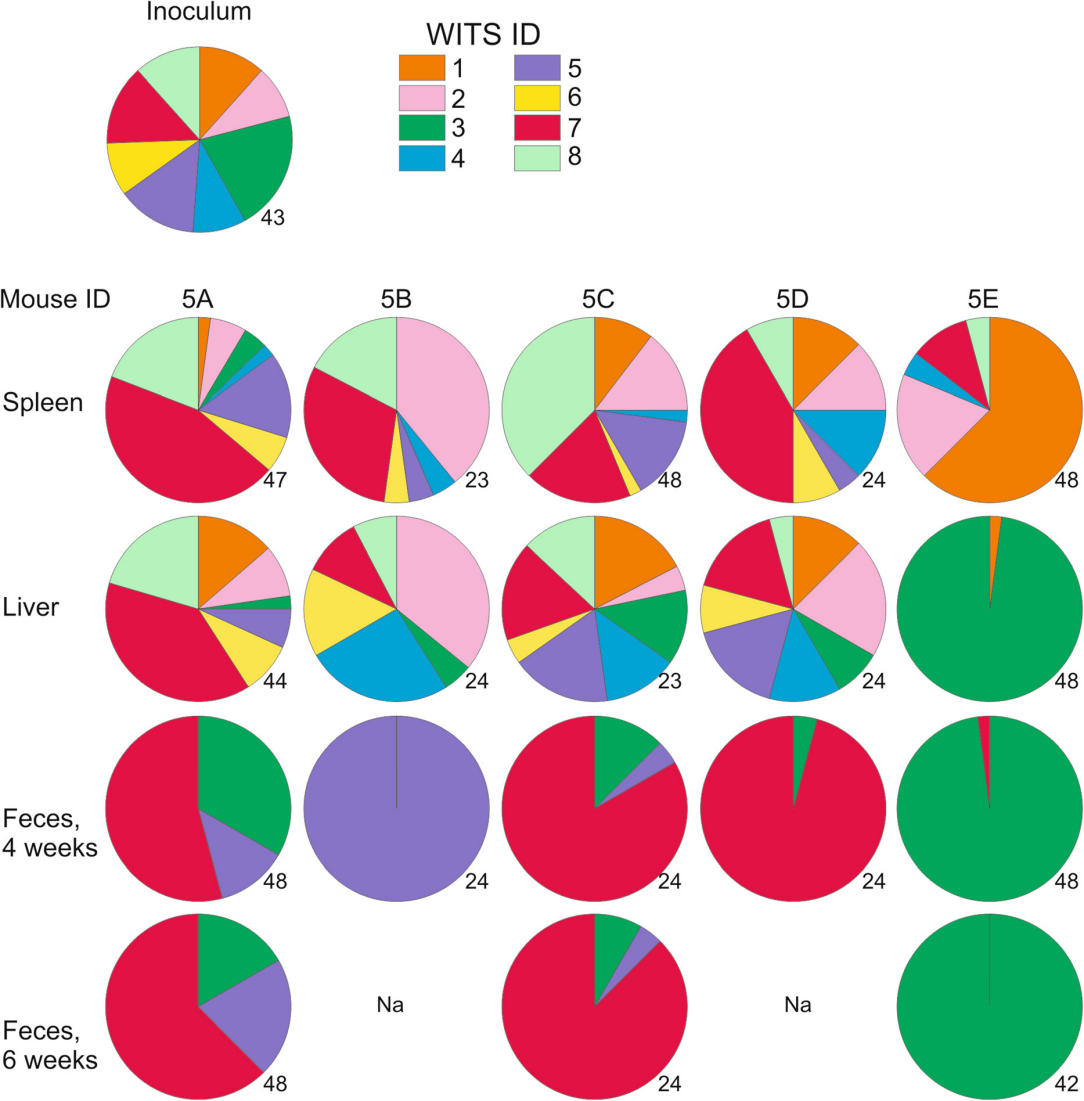
Dominant Salmonella clones emerge during chronic infections. Five mice were infected I.P. with 10^4^ *S*. Typhimurium consisting of an equal mixture of eight WITS strains (the inoculum). WITS distribution in indicated samples was determined by a PCR/sequencing strategy. Fecal samples were collected after 4 to 6 weeks of infection, as indicated. All systemic samples were collected after 6 weeks, except for samples from mice 5B and 5D which were euthanized after 4 weeks. Pie charts depict the relative abundance of each WITS strain in different samples. Each pie chart contains the WITS profile of the indicated sample. Above pie charts, the mouse IDs from which the sample was collected are indicated. To the left of pie charts, the respective samples are denoted. The number to the bottom right of each pie chart denotes the number of individual clones analyzed

To identify potential dominant clones the distribution of WITS in *S*. Typhimurium clones isolated from fecal, spleen and liver samples were determined using a PCR and sequencing based strategy (Fig. 1). All fecal samples had clear dominant clones comprising 65 to 100 % of isolates. Dominant clones were also found at systemic sites. In some mice, the same WITS was dominant in more than one sample whereas in other mice, dominant WITS differed between isolation sites. Together, these results are consistent with a previous report on the infection dynamics of *S*. Typhimurium during chronic infections [12]. Interestingly, three mice (5A, 5C and 5D) exhibited similar WITS distribution patterns in their feces (Fig. 1) indicating potential transmission of clones between mice via the fecal-oral route.

### Dominant clones have acquired potential patho-adaptive mutations

To investigate if the emergence of dominant clones was a result of stochastic infection dynamics as previously reported or due to genetic adaptation, representative clones exhibiting more than 50 % dominance were selected for whole genome sequencing using the Illumina platform. Specifically, the dominant fecal clones of mice 5A, 5B, 5C, 5D, and 5E, and the dominant clones of the liver and spleen samples of mouse 5E were selected for sequencing. As a control, the parental ST4/74 strain used for strain construction was also sequenced. In all selected isolates, single nucleotide polymorphisms (SNPs) were detected (Table 2). In most isolates only one SNP was detected, whereas in the splenic isolate of mouse 5E and the fecal isolates from mice 5B, two SNPs were detected (Table 2). All detected SNPs were confirmed by PCR and Sanger sequencing. In mouse 5E the same non-synonymous SNP in *ptsN*, encoding a regulator of SPI2 gene expression [13], was identified in both the liver and fecal isolates of this mouse. This could indicate that the mutation occurred at one of these sites and then spread to the other site within the host. Interestingly, this mouse had a different dominant clone in the spleen sample containing two distinct mutations; a single base deletion (T) in the promoter region of *yicE*, encoding a *xanP* homologue, the other is a synonymous SNP in ST474_0057. These results point to niche specific adaptation of *S*. Typhimurium within the same host. In the fecal samples from mice 5A and 5C, the exact same non-synonymous SNP in *kdgR*, encoding a regulator of metabolic genes [14], was detected. This indicates that this SNP occurred in one of the mice and was subsequently transmitted to the other mouse.

**Table 2:** Whole genome sequencing results

SRA accession number^a^	Mouse ID	Sample	Clone	WITS ID	WITS prevalence	SNP^b^	Coordinates in ST4/74 genome^c^	Gene affected and amino acid effect^d,e^
SAMN04230908	5A	Feces, 6 weeks	2A	7	63 %	G- > T	1895850	*kdgR*, V61L
SAMN04230909	5B	Feces, 4 weeks	1A	4	>96 %	A- > G G- > A	22493 2578136	Synonymous, located in STM474_0020 Synonymous, located in *maeB*
SAMN04230910	5C	Feces, 6 weeks	2A	7	88 %	G- > T	1895850	*kdgR*, V61L
SAMN04230911	5D	Feces, 4 weeks	1A	7	96 %	G- > T	1895850	*kdgR*, V61L
SAMN04230912	5E	Spleen	1A	1	63 %	Deletion, T A- > G	3962521 63854	Promoter region of *yicE* Synonymous, located in STM474_0057
SAMN04230913	5E	Liver	1A	3	98 %	C- > A	3507750	*ptsN*, S21R
SAMN04230914	5E	Feces, 6 weeks	1A	3	>98 %	C- > A	3507750	*ptsN*, S21R

^a^Short Read Archive database accession numbers for the raw sequencing reads

^b^Single nucleotide polymorphisms

^c^Accession numbers NC_016857, CP002488-CP002490 [25]

^d^Genes are denoted by their common name or by their STM474 gene number

^e^Amino acids are denoted by their single letter code

In conclusion, during this relatively short-term experimental chronic infection numerous mutations are detected in isolated clones. Most of these clones are associated with the intestinal environment; however, in one mouse mutations are also detected in isolates from systemic organs. Furthermore, in this mouse distinct mutations are detected in the liver- and spleen-clones, respectively, providing evidence for niche specific adaptation within the same host with limited migration between organs. Finally, the sequencing results also reveal that three mice are colonized by the same clone in the intestines implying transmission of the dominant clone between these mice via the fecal-oral route.

### The ptsN-SNP clone is present and dominant in the fecal sample of mouse 5E at four weeks post infection

**Fig. 2:**
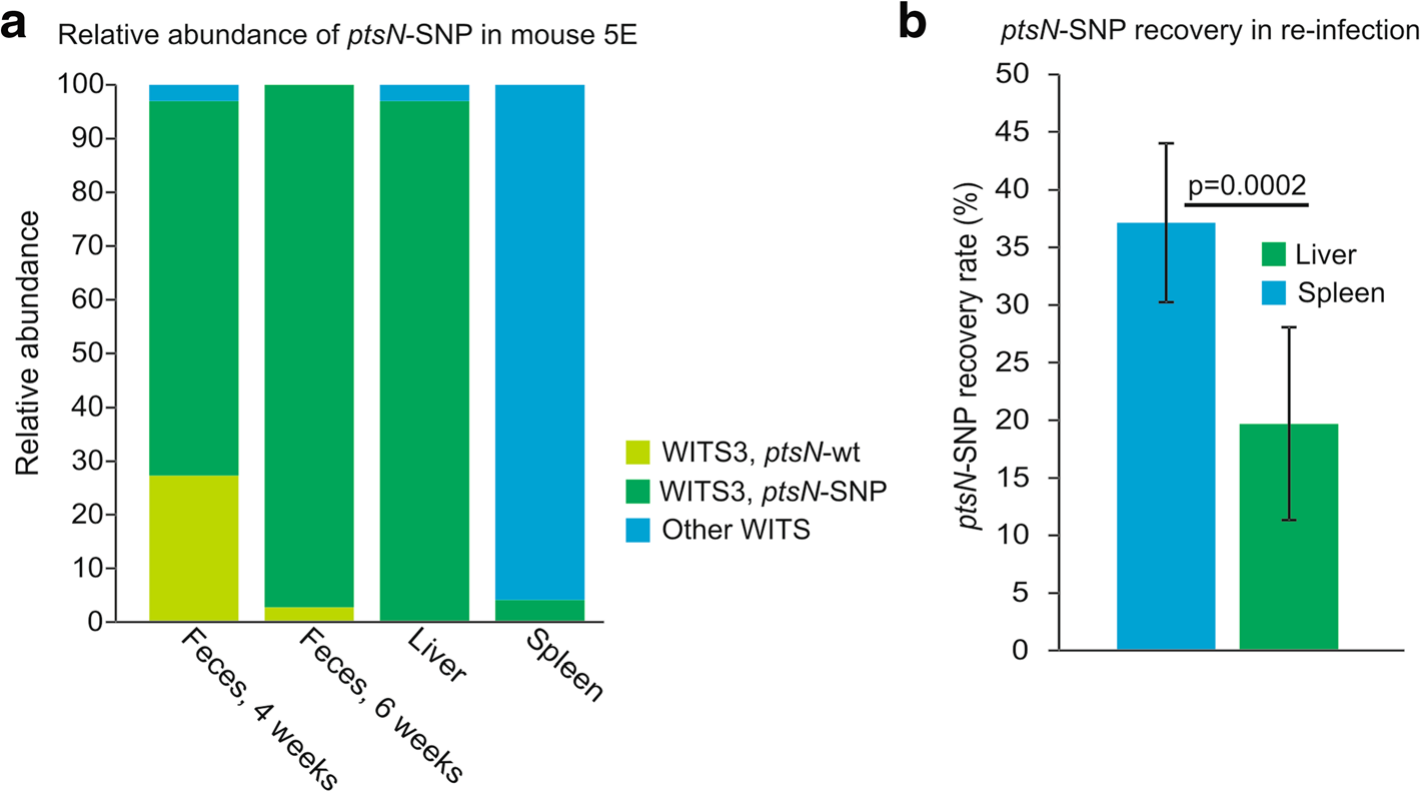
Prevalence of the *ptsN*-SNP mutation. **a** Analysis of the *ptsN* locus of all WITS3 clones isolated from mouse 5E. Bars depict the relative abundance of the WITS3 (*dark green* and *light green*) in comparison with all other WITS (*blue*), and the relative occurrence of the *ptsN*-SNP allele (*dark green*) versus the *ptsN*-wt (*light green*) allele within the WITS3 population of the indicated samples. **b** Relative recovery of the *ptsN*-SNP allele after re-infection (I.P.) of ten mice with the *ptsN*-wt-WITS1, *ptsN*-SNP-WITS3 and an isogenic Δ*ptsN* mutant strain. Bars depict the relative recovery of the *ptsN*-SNP clone in the spleen and liver after 5 weeks of infection. Error bars depict standard deviation. Recovery of the *ptsN*-SNP from the liver sample is significantly lower (*p* = 0.0002) than from the spleen sample as determined by a two-sample t-test with unequal variances

In mouse 5E a mutation in *ptsN* was detected in both the fecal and liver isolate. The mutation results in an amino acid substitution from Serine to Arginine at position 21 in the PtsN protein. PtsN is a conserved protein and have been implicated in potassium homeostasis in *E. coli* [15, 16]. In *S*. Typhimurium, PtsN has been shown to be essential for virulence during acute infections. Further studies revealed that PtsN functions as a repressor of SPI2 gene expression by direct interaction with SsrB [13], a response regulator and primary activator of SPI2 virulence gene expression [17]. The role of PtsN in chronic infections have not been reported, however, SPI2 encoded virulence factors are essential for both acute and chronic infections [5, 18], suggesting the possibility that PtsN may also be important during chronic infections. To investigate the level of dominance of this clone in the 6-weeks fecal sample and the liver sample, and to determine the temporal occurrence of the *ptsN*-SNP, all *S*. Typhimurium clones with the same WITS (3) isolated from this mouse was examined by sequencing the *ptsN* locus. This revealed that all (*n* = 33) WITS3 clones of the liver sample, 97 % of the 6 week fecal sample (*n* = 36), and 69 % of the 4 week fecal sample (*n* = 33) contained the *ptsN*-SNP. The remaining WITS3 clones carried the wt *ptsN* allele (Fig. 2a). Thus, the mutation occurred prior to 4 weeks post infection, most likely in the liver. The temporal progression in the feces from 4 to 6 weeks post infection together with the level of dominance of the clone indicates that the *ptsN*-SNP confers an advantage for survival at either or both of these intra host niches, the liver and intestines, respectively. Interestingly, the two WITS3 clones isolated from the spleen also carried the *ptsN*-SNP, showing that the clone had been circulating in the organs; however, it did not become dominant in the spleen during the course of this infection. Rather, the spleen harbored a distinct dominant clone containing two other mutations (Table 2).

To investigate the role of *ptsN* during chronic infections and to determine if the *ptsN*-SNP confers any advantages for survival inside the host as suggested by the dominance of the clone, two new groups of 5 mice each were infected IP to establish chronic infections with a 1:1:1 mixture of ST4/74-WITS1 (wt-WITS1), the *ptsN*-SNP-WITS3-clone and an isogenic strain deleted for *ptsN* (*ΔptsN*). The infection was allowed to proceed for 5 weeks and *S*. Typhimurium bacteria were isolated from fecal samples every week and from livers and spleens upon termination. Isolated bacteria were genotyped (wt-WITS1, *ptsN*-SNP-WITS3 or *ΔptsN*) using a PCR/sequencing strategy. Only two mice were colonized by *S*. Typhimurium in the intestines prior to 3 weeks post infection (data not shown). This is in accordance with the first experiment (Table 1). At no point during the infection, from either the fecal or the organ samples of all 10 mice, was the *ΔptsN* strain isolated. This signifies that similar to acute infections, *ptsN* is essential for chronic infections. Only wt-WITS1 clones were isolated from the fecal samples with the exception of mouse 5 of group 2 where the fecal sample was dominated by the *ptsN*-SNP clone throughout the experiment (data not shown). In the spleen, the *ptsN*-SNP clone was present in an average of 37 %+/−7.% of analyzed clones, and in the liver it was significantly less prevalent with a 20 %+/−8.4 % recovery rate (*p* = 0.0002) (Fig. 2b). These results could suggest that the *ptsN*-SNP is less fit than the wt-WITS1 in both the spleen and liver during this infection and does not corroborate the observation from the original experiment from which the *ptsN*-SNP clone was isolated. Further investigations are required to elucidate the specific roles of PtsN during chronic infections and the consequence of the *ptsN*-SNP mutation.

### The kdgR-mutation originated in mouse 5D at an early time-point post infection

**Fig. 3:**
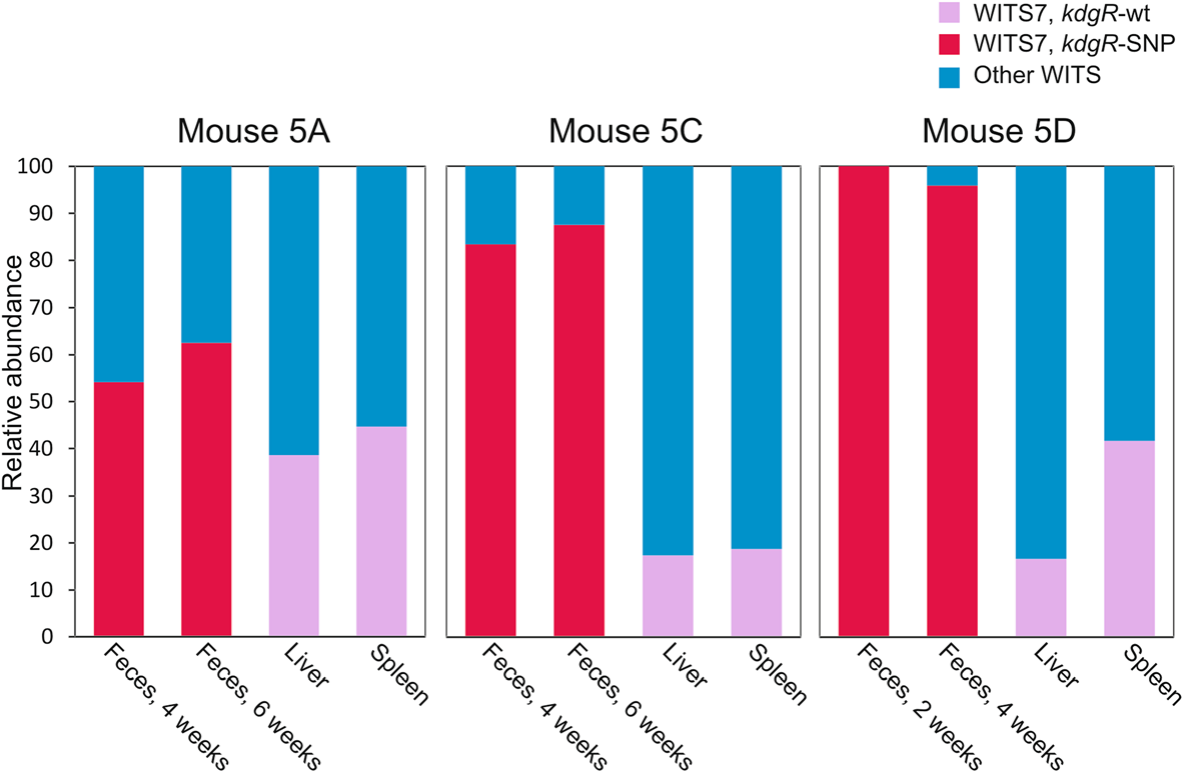
The *kdgR*-SNP mutation is dominant in fecal samples, but not present in systemic samples of mice 5A, 5C, and 5D. Analysis of the *kdgR* locus of all WITS7 clones isolated from mice 5A, 5C and 5D. Each box depicts sample from the mouse indicated above the box. Bars depict the relative abundance of the WITS7 (*red* and *purple*) in comparison with all other WITS (*blue*), and the relative occurrence of the *kdgR*-SNP allele (*red*) versus the *kdgR*-wt (*purple*) allele within the WITS7 population of the indicated samples

In three mice, (5A, 5C and 5D) a similar WITS-pattern was observed in the fecal samples, indicating transmission of clones between these mice. This was further supported by the sequencing data revealing that the dominant WITS7 clone of the fecal samples of mouse 5A and 5C contained the exact same SNP in *kdgR*, a regulator of metabolic genes (Table 2). The mutation results in an amino acid substitution from Valine to Leucine at position 61 in the KdgR protein. To further investigate the possibility that this clone originated in one of the mice and was subsequently transmitted to the others, the *kdgR* locus was sequenced in all WITS7 clones isolated from these three mice exhibiting similar WITS patterns (Fig. 3). In mouse 5A and 5C, the *kdgR*-SNP allele was dominant in both the 4 weeks and 6 weeks samples; however, it was not recovered from any systemic sites. In mouse 5D, which exhibited an early intestinal colonization with high *S*. Typhimurium numbers in the feces after 2 weeks (Table 1), sequencing of the *kdgR*-locus revealed that the *kdgR*-SNP allele was present and completely dominant in the feces at both 2 and 4 weeks post infection. At 4 weeks, mouse 5D was euthanized. Similar to mouse 5A and 5C, only the *kdgR*-wt allele was identified in the organ samples of mouse 5D. Together these results show that the *kdgR*-SNP arose in the intestinal environment prior to 2 weeks post infection, that the mutation most likely originated in mouse 5D and that the clone was subsequently transmitted to mouse 5A and 5C via the fecal-oral route. Interestingly, sequencing of the *kdgR* locus of WITS7 clones isolated from the other mice of the cage revealed that it was only mouse 5A and 5C that had been colonized by the *kdgR*-SNP clone from mouse 5D (data not shown).

### The kdgR-SNP mutant exhibits rapid intestinal colonization upon re-infection

**Fig. 4:**
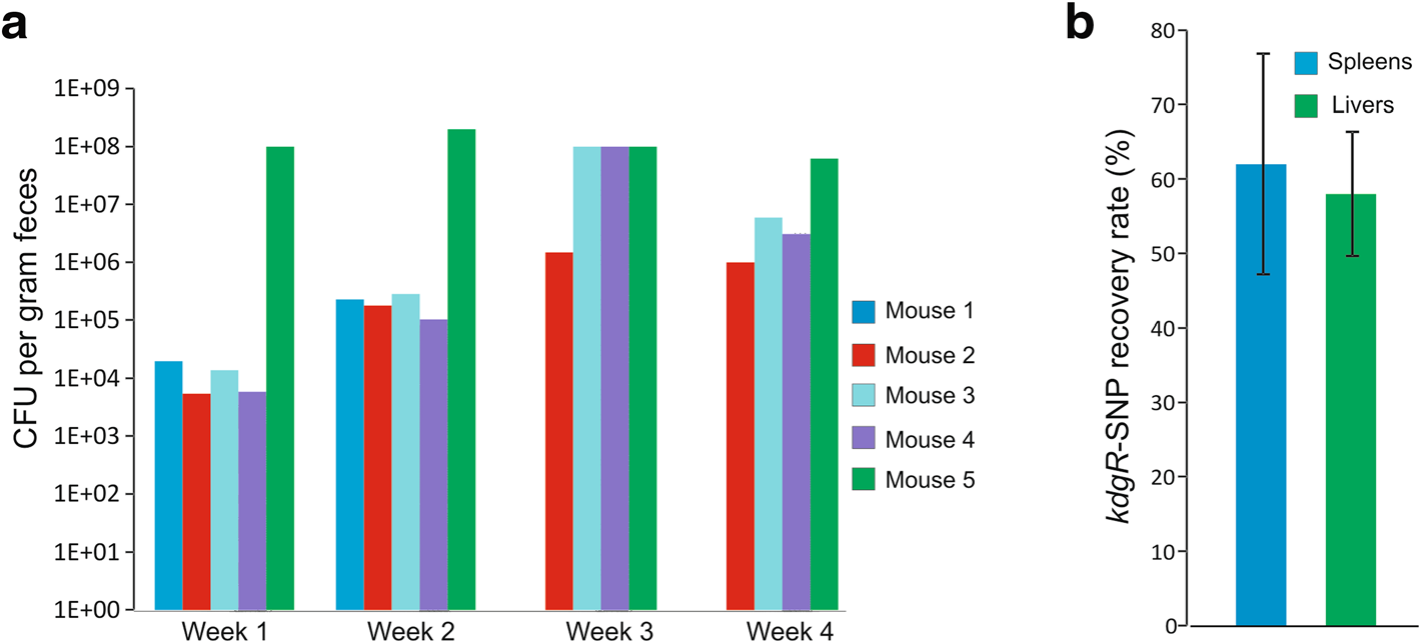
The *kdgR*-SNP mutant exhibits rapid intestinal colonization in re-infection experiment. **a** CFU of *S*. Typhimurium bacteria isolated from the fecal samples of individual five mice following infection (I.P) with an equal mixture of *kdgR*-wt-WITS1 and *kdgR*-SNP-WITS7 bacteria. Mouse 1 was euthanized after 2 weeks. **b** Relative recovery of the *kdgR*-SNP allele after re-infection (I.P.) of five mice with the *kdgR*-wt-WITS1 and the *kdgR*-SNP-WITS3. Bars depict the relative recovery of the *kdgR*-SNP clone in the spleen and liver after 4 weeks of infection. Error bars depict standard deviation

To investigate the role of *the kdgR-SNP* during chronic infections and to determine if the *kdgR*-SNP conferred any advantages for intestinal colonization as suggested by the dominance of the clone, a new group of 5 mice was infected IP to establish chronic infections with a 1:1 mixture of ST4/74-WITS1 (wt-WITS1) and the *kdgR*-SNP-WITS7-clone. The infection was allowed to proceed for 4 weeks and *S*. Typhimurium bacteria were isolated from fecal samples every week and from livers and spleens upon termination. Isolated bacteria were genotyped (wt-WITS1 or *kdgR*-SNP-WITS7) using a PCR/sequencing strategy. Within 1 week, all mice were colonized in the intestines by the *kdgR*-SNP-clone (Fig. 4a). This is highly unusual given the route of infection (IP) and that the mice were not pretreated with streptomycin. In particular one mouse, mouse 5, had a high number of *S*. Typhimurium bacteria in the feces, 10^8^ *S*. Typhimurium bacteria per gram feces. This high number of *S*. Typhimurium bacteria is characteristic for a so-called super shedder [19]. In the following weeks, the remaining mice increased in fecal CFU and this sustained throughout the experiment. Interestingly, the wt-WITS1 clone was never recovered from the fecal samples of these mice, pointing to the very successful intestinal colonization of the *kdgR*-SNP-clone. Mouse 1 had to be terminated after 2 weeks due to symptoms. After 4 weeks of infection, remaining mice were euthanized and systemic organs were examined for the presence of *S*. Typhimurium bacteria. Livers and spleens had an almost equal mixture of wt-WITS1 and *kdgR*-SNP clones signifying that the *kdgR*-SNP-clone is able to establish a systemic infection at similar levels as the wt (Fig. 4b).

### The kdgR-SNP mutant has constitutive repression of its target gene kdgK

The KdgR-protein is a repressor of *kdgK* and *kdgT*, two metabolic genes of the KDG-branch involved in transport and catabolism of 2-keto-3-deoxygluconate (KDG) [14]. KDG can be imported by KdgT or result from degradation of starch or glucoronate. KDG is then converted to KDG-6-phosphate (KDGP) by the KdgK kinase. At this point, the KDG-branch converges with the Entner-Doudoroff (ED) pathway catabolizing gluconate-6-phosphate to first KDGP and then to pyruvate and D-glyceraldehyde-3-phosphate by the KDGP aldolase encoded by *eda*. KdgR also represses expression from one of three promoters controlling the *eda* gene, encoding a central enzyme of the ED-pathway. However, this effect of KdgR on *eda* expression is negligible [20]. The ED-pathway functions as an alternative to the Embden-Meyerhof-Parnas glycolytic pathway [21], and in *E. coli* the ED-pathway is essential for intestinal colonization of mice [22].

**Fig. 5:**
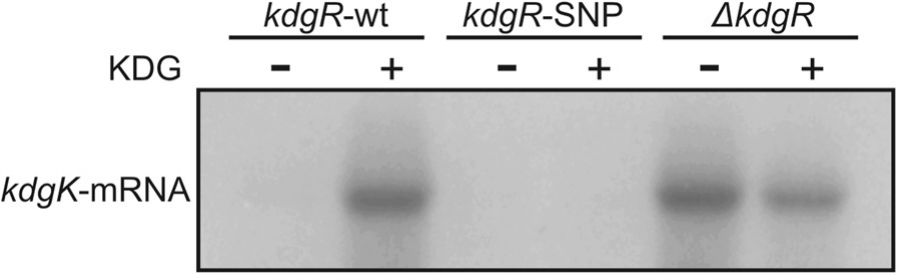
The *kdgR*-SNP elicits constitutive repression of its target gene *kdgK*. The wt, the kdgR-SNP and a Δ*kdgR* strain were grown in M9 minimal media at 37 °C with shaking until mid-logarithmic phase. At this point a sample from each strain was collected and the KdgR inducer KDG was added to all cultures. Cultures were re-incubated for an hour and a sample from each culture was collected. Total RNA was extracted from all samples, and the level of the *kdgK* transcript was assessed by northern blot analysis using a radioactive probe specific for *kdgK*

KdgR negatively affects expression of its target genes by binding to the promoter regions. Relieve of repression is achieved by induction with the substrate KDG [14]. The mutation identified in the *kdgR*-SNP is located in the DNA-binding HTH domain potentially affecting the DNA-binding and regulatory activity of KdgR. To investigate this possibility, expression of one of its target genes, *kdgK*, was measured in RNA extracted from the wt, the isogenic *kdgR*-SNP mutant and an isogenic strain where the *kdgR* gene had been deleted (Δ*kdgR*). Cultures were grown in M9 minimal media and samples for total RNA extraction were derived from exponential cultures grown with and without the KdgR-inducer KDG. Expression of *kdgK* was monitored with northern blotting using a probe specific for the *kdgK*-transcript. In the wt, expression of *kdgK* was not detectable during growth in M9, however, upon addition of KDG to the cultures, expression was strongly induced (Fig. 5). In a strain lacking *kdgR* (Δ*kdgR*), expression of *kdgK* was constitutively high independently of the presence of the inducer KDG. In contrast, in the *kdgR*-SNP mutant, expression was barely detectable and not induced by addition of the inducer KDG. These data indicate that the SNP-mutation found in the *kdgR* gene alters the activity of KdgR making it un-responsive to induction by KDG and provide evidence for altered metabolic regulation of this clone. Specifically, it appears that expression of genes of the KDG-branch is constitutively repressed by the KdgR-SNP protein. Interestingly, in *E. coli*, it has been shown that accumulation of KDGP, the product of KdgK kinase activity, is toxic leading to growth arrest [23], providing a possible explanation for the observed selective advantage of the *kdgR*-SNP during intestinal colonization. Further investigations are required to elucidate the metabolic consequences of the *kdgR*-SNP and the underlying molecular mechanisms of its altered regulatory activity.

## Conclusions

In the present study, the level of genetic adaptation was investigated during a relative short S. Typhimurium infection of a mouse model of chronic infections. This revealed that even within a very short time frame (2 to 4 weeks post infection) *S*. Typhimurium acquires distinct patho-adaptive mutations conferring selective advantage during chronic infections. Together with the recent availability of whole genome sequencing as a common tool in molecular microbiology this study serves as a proof-of-concept for further investigations into the molecular and evolutionary mechanisms of host-adaptation. For example, data presented here point to that even one SNP in the genome of *S*. Typhimurium confers a clear advantage for intestinal colonization and growth with indications of increased transmission as a consequence. Further investigations of the effect of this SNP will provide insight into the metabolism of *S*. Typhimurium during intestinal colonization. Other mutations identified were synonymous or located at intergenic regions. Although, the effect of these mutations is more difficult to infer from the sequence alone, they may still provide selective advantage by altering the regulation and expression of neighboring genes [24]. In one mouse, distinct adapted clones were isolated from the liver and spleen, respectively, pointing to potential within-host niche specific adaptation. Overall, the results presented here provide novel insight into host-pathogen interactions and present a new approach for studying this at the molecular level. Of note, the rapid occurrence of adaptive mutations reported here should be taken into account in future studies investigating long-term bacteria-environmental interactions.

## Methods

### Bacterial strains and growth conditions

Bacterial strains and plasmids are listed in Table 3. *S.* Typhimurium ST4/74 was used as wild-type strain in all experiments. This strain has been described previously [25, 26] and its virulence is well defined [27, 28]. *S.* Typhimurium strains were maintained in LB media. For solid medium, 1.5 % agar was added to give LB agar plates. Kanamycin (50 μg ml^−1^) or carbenicillin (50 μg ml^−1^) was added as required. Prior to all experiments bacteria were grown for 16 h, 200 rpm, 37 °C in LB or M9 (2 mM MgSO_4_, 0.1 mM CaCl_2_, 0.4 % glucose, 8.5 mM NaCl, 42 mM Na_2_HPO_4_, 22 mM KH_2_PO_4_, 18.6 mM NH_4_Cl) media as indicated. *Escherichia coli* Top10 competent cells (Invitrogen) were used for DNA cloning and were grown in LB media or on LB agar plates at 37 °C with appropriate antibiotics.

**Table 3:** Strains and plasmids used in the study

Strains	Relevant genotype	Reference
ST4/74	*S*.Typhimurium strain ST4/74. Reference strain.	[25]
LJ584	*S*.Typhimurium strain ST4/74, WITS1, kan^R^	This study
LJ585	*S*.Typhimurium strain ST4/74, WITS2, kan^R^	This study
LJ586	*S*.Typhimurium strain ST4/74, WITS3, kan^R^	This study
LJ587	*S*.Typhimurium strain ST4/74, WITS4, kan^R^	This study
LJ588	*S*.Typhimurium strain ST4/74, WITS5, kan^R^	This study
LJ589	*S*.Typhimurium strain ST4/74, WITS6, kan^R^	This study
LJ590	*S*.Typhimurium strain ST4/74, WITS7, kan^R^	This study
LJ591	*S*.Typhimurium strain ST4/74, WITS8, kan^R^	This study
LJ684	*S*.Typhimurium strain ST4/74, Δ*kdgR*, kan^R^	This study
LJ678	*S*.Typhimurium strain ST4/74, Δ*ptsN*, kan^R^	This study
Top10	*E. coli* cloning strain	Invitrogen
Plasmids		
pCR®2.1-TOPO	TOPO-cloning vector, amp^R^, kan^R^	Invitrogen
pACYC177	Cloning vector, amp^R^, kan^R^	[32]
pKD46	Plasmid with λ-Red recombinase expressed from arabinose inducible promoter	[29]
pKD4	Template plasmid for λ-Red mutagenesis, kan^R^	[29]

### Construction of tagged strains

All primers used in the study can be found in Additional file 1: Table S1. To construct 8 individually tagged strains the kanamycin gene from pACYC177 were used as template in PCR reactions with 8 individual forward primers (Tag1kanfwd, Tag2kanfwd, Tag3kanfwd, Tag4kanfwd, Tag5kanfwd, Tag6kanfwd, Tag7kanfwd, Tag8kanfwd), each with a unique sequence tag on the linker and one common reverse primer (Tagkanrev2). The 8 PCR products were TOPO-cloned into the pCR®2.1-TOPO vector from Invitrogen according to the manufactures recommendations. A fragment containing an individual 40-bp tag and the kanamycin resistance cassette was amplified from the individually tagged TOPO-vectors, using the primers Tag1malXYfwd, Tag2malXYfwd, Tag3malXYfwd, Tag4malXYfwd, Tag5malXYfwd, Tag6malXYfwd¸ Tag7malXYfwd¸ Tag8malXYfwd, and Tagsmalxyrev2. Approximately 1 μg of each linear PCR product was used for integration into *malXY* pseudogene locus of the chromosome of ST4/74 *S*. Typhimurium using the Lambda Red method as described previously [29]. Transformants were selected by plating onto selective media. Correct integration of the tag-sequence and the kanamycin gene into the *malXY* locus was confirmed using a PCR strategy with the primers malXYcon and Tagkanrev2. The tags were then reintroduced into a wild-type *S*. Typhimurium ST4/74 background via transduction with phage P22 HT105/1 *int*201 using previously described protocols. Gene deletions and concomitant insertions of an antibiotic resistance cassette were constructed using Lambda Red mediated recombination as described elsewhere [29]. All constructs were verified by PCR and re-introduced into a wt ST4/74 background via P22 phage transduction as previously described [27]. 

### Infections of mice

Seven weeks old female 129X1/SvJ mice, 16–18 g, were used to establish chronic *Salmonella* infections. Ten mice were purchased from Jackson Laboratories. Upon arrival, mice were randomly distributed into two groups of five mice each. Throughout mice had unlimited access to food and water, and cages were supplied with bedding for environmental enrichment. For individual assessment, all mice were earmarked. The infections were carried out by the section for experimental medicine (AEM) at Copenhagen University, where they have specialized facilities for animal housing and expertise for animal handling. To prepare the inoculums each WITS strain were grown for 16 h, 200 rpm at 37 °C in LB media. The 8 WITS strains were mixed 1:1:1:1:1:1:1:1 before the infection to give a challenge dose of 10^4^ bacteria in total. 5 mice caged in the individually ventilated cage (IVC) were inoculated via the intraperitoneal route. A control group of 5 mice was administered physiological saline. The route of infection was chosen as the aim of the study was to establish an asymptomatic systemic infection. The exact bacterial number and ratio between individually tagged strains was determined by plating and by a PCR based sequencing approach as described below. Mice were manually inspected for symptoms of distress every 8 h during the first week post infection, and every 12 h for the remainder of the experiment. Over the course of the infection, fecal samples were collected from individual mice and *S*. Typhimurium bacteria were recovered and enumerated after plating a dilution series on to LB agar containing kanamycin. Mice were killed at 6 weeks post infection by cervical dislocation. In case a mouse displayed symptoms of illness, it was euthanized prior to this time point. The spleens and livers were removed aseptically and bacteria were recovered and enumerated after plating a dilution series on to LB agar containing kanamycin. Up to 96 individual *S*. Typhimurium clones from each sample (fecal and organ) were collected and frozen in glycerol stocks at −80 °C. Re-infections were carried out as described above in groups of five mice in one group. For re-infections isolated adapted clones were mixed 1:1 with the wild type and mice were infected IP with a challenge dose of 10^4^ bacteria in total. Re-infections were terminated after 4 weeks of infections.

### Ethics statement

All mouse experiments were reviewed and approved by the Copenhagen University animal experimentation unit and conducted with permission from the Animal Experiments Inspectorate (http://www.dyreforsoegstilsynet.dk) under license number 2013-15-2934-00761 in accordance with Danish law LBK 474 af 15/05/2014 (Animal experimentation and welfare act).

### PCR and sequencing of WITS clones

Distribution of WITS in the inoculums and isolated clones recovered from fecal samples and systemic organs after infections were determined by a PCR based sequencing approach. For each sample the WITS was determined in 20 to 48 individual clones by PCR amplifying the region containing the WITS followed by Sanger sequencing of individual PCR products. Primer sequences (Tagseqfwd and Tagseqrev) used for PCR amplification of the WITS region can be found in Additional file 1: Table S1.

### Extraction of chromosomal DNA and whole genome sequencing of dominant clones

Bacterial cells were grown overnight in 5 ml LB at 37 °C with 200 rpm. The next day cultures were harvested and DNA was extracted using the ChargeSwitch gDNA Bacteria Kit from Invitrogen according to the manufacturer’s recommendation. Samples were stored at −20 °C. DNA was analyzed by Nanodrop 1000 from Thermo Fischer and shipped to BGI or Macrogen for Whole Genome Sequencing using the Illumina HiSeq 2000 platform with 100 bp paired end reads. Paired-end reads were mapped on the ST4/74 genome sequence (NCBI NC_016857, CP002488-CP002490) using the Geneious Alignment Tool giving a raw coverage depth of approximately 100–200 fold. Variant base calling relative to ST4/74 was performed with the Geneious work package. Sequence reads from all isolates are deposited in the Short Read Archive under accession number SRP070821 (accession numbers for individual samples are provided in Table 2). SNPs supported by 95–100 % of the reads, and all single nucleotide deletions and insertions (indels) were re-examined by Sanger capillary sequencing of PCR products amplified using specific primers (Additional file 1: Table S1) flanking the regions of interest.

### Extraction of RNA and northern blotting

Cells were grown in M9 for 16 h, 200 rpm at 37 °C. The next day all cultures were diluted 100-fold into fresh M9 an incubated at 37 °C with shaking at 200 rpm. Growth was monitored by OD_600_ measurements every hour. At OD_600_ ~ 0.4 +/−0.1, 1 ml aliquots were harvested from each culture and immediately frozen and stored at −80 °C. At this point 2-keto-3-deoxygluconate (KDG) was added to all cultures at a final concentration of 5.6 mM (0.1 mg ml^−1^). The cultures were re-incubated for 1 h and 1 ml aliquots were again harvested from all cultures. Cells were lysed mechanically using the FastPrep system (Bio101; Q-biogene), and total RNA was isolated as described previously [30] using the RNeasy mini kit (QIAGEN, Valencia, Calif.) according to the manufacturer's instructions. Total RNA was quantified by Nanodrop 1000 from Thermo Fischer and 5 μg of RNA of each preparation was loaded onto a 1 % agarose gel and separated in 10 mM sodium phosphate buffer. RNA was transferred to a positively charged nylon membrane (Boehringer Mannheim) by capillary blotting. Hybridization was performed according to [31] using a gene-specific *kdgK* probe that had been labeled with [^32^P]dCTP using the Ready-to-Go DNA-labeling beads from Amersham Biosciences. Internal fragments of the *kdgK* gene were used as template in the labeling reaction. All steps were repeated in three independent experiments giving similar results.

### Statistical analysis

Significant differences were determined using the 2 sample t-test with unequal variances. Error bars represent standard deviations.

## Additional file

Additional file 1: Table S1. List of primers used in the study. (DOCX 17 kb)

## References

[1] Haraga A, Ohlson MB, Miller SI (2008). Salmonellae interplay with host cells. Nat Rev Microbiol.

[2] Levine MM, Black RE, Lanata C (1982). Precise estimation of the numbers of chronic carriers of Salmonella typhi in Santiago, Chile, an endemic area. J Infect Dis.

[3] Monack DM, Bouley DM, Falkow S (2004). Salmonella typhimurium persists within macrophages in the mesenteric lymph nodes of chronically infected Nramp1+/+ mice and can be reactivated by IFNgamma neutralization. J Exp Med.

[4] Shea JE, Beuzon CR, Gleeson C, Mundy R, Holden DW (1999). Influence of the Salmonella typhimurium pathogenicity island 2 type III secretion system on bacterial growth in the mouse. Infect Immun.

[5] Lawley TD, Chan K, Thompson LJ, Kim CC, Govoni GR, Monack DM (2006). Genome-wide screen for Salmonella genes required for long-term systemic infection of the mouse. PLoS Pathog.

[6] Rau MH, Hansen SK, Johansen HK, Thomsen LE, Workman CT, Nielsen KF (2010). Early adaptive developments of Pseudomonas aeruginosa after the transition from life in the environment to persistent colonization in the airways of human cystic fibrosis hosts. Environ Microbiol.

[7] Ford CB, Lin PL, Chase MR, Shah RR, Iartchouk O, Galagan J (2011). Use of whole genome sequencing to estimate the mutation rate of Mycobacterium tuberculosis during latent infection. Nat Genet.

[8] Yang L, Jelsbak L, Marvig RL, Damkiaer S, Workman CT, Rau MH (2011). Evolutionary dynamics of bacteria in a human host environment. Proc Natl Acad Sci U S A.

[9] Grant AJ, Restif O, McKinley TJ, Sheppard M, Maskell DJ, Mastroeni P (2008). Modelling within-host spatiotemporal dynamics of invasive bacterial disease. PLoS Biol.

[10] Blackwell JM, Searle S, Goswami T, Miller EN (2000). Understanding the multiple functions of Nramp1. Microbes Infect.

[11] Monack DM (2012). Salmonella persistence and transmission strategies. Curr Opin Microbiol.

[12] Lam LH, Monack DM (2014). Intraspecies competition for niches in the distal gut dictate transmission during persistent Salmonella infection. PLoS Pathog.

[13] Choi J, Shin D, Yoon H, Kim J, Lee CR, Kim M (2010). Salmonella pathogenicity island 2 expression negatively controlled by EIIANtr-SsrB interaction is required for Salmonella virulence. Proc Natl Acad Sci U S A.

[14] Pouyssegur J, Stoeber F (1974). Genetic control of the 2-keto-3-deoxy-d-gluconate metabolism in Escherichia coli K-12: kdg regulon. J Bacteriol.

[15] Lee CR, Cho SH, Yoon MJ, Peterkofsky A, Seok YJ (2007). Escherichia coli enzyme IIANtr regulates the K+ transporter TrkA. Proc Natl Acad Sci U S A.

[16] Luttmann D, Heermann R, Zimmer B, Hillmann A, Rampp IS, Jung K (2009). Stimulation of the potassium sensor KdpD kinase activity by interaction with the phosphotransferase protein IIA(Ntr) in Escherichia coli. Mol Microbiol.

[17] Garmendia J, Beuzon CR, Ruiz-Albert J, Holden DW (2003). The roles of SsrA-SsrB and OmpR-EnvZ in the regulation of genes encoding the Salmonella typhimurium SPI-2 type III secretion system. Microbiology.

[18] Hensel M, Shea JE, Gleeson C, Jones MD, Dalton E, Holden DW (1995). Simultaneous identification of bacterial virulence genes by negative selection. Science.

[19] Lawley TD, Bouley DM, Hoy YE, Gerke C, Relman DA, Monack DM (2008). Host transmission of Salmonella enterica serovar Typhimurium is controlled by virulence factors and indigenous intestinal microbiota. Infect Immun.

[20] Murray EL, Conway T (2005). Multiple regulators control expression of the Entner-Doudoroff aldolase (Eda) of Escherichia coli. J Bacteriol.

[21] Peekhaus N, Conway T (1998). What's for dinner?: Entner-Doudoroff metabolism in Escherichia coli. J Bacteriol.

[22] Chang DE, Smalley DJ, Tucker DL, Leatham MP, Norris WE, Stevenson SJ (2004). Carbon nutrition of Escherichia coli in the mouse intestine. Proc Natl Acad Sci U S A.

[23] Fuhrman LK, Wanken A, Nickerson KW, Conway T (1998). Rapid accumulation of intracellular 2-keto-3-deoxy-6-phosphogluconate in an Entner-Doudoroff aldolase mutant results in bacteriostasis. FEMS Microbiol Lett.

[24] Marvig RL, Damkiaer S, Khademi SM, Markussen TM, Molin S, Jelsbak L (2014). Within-host evolution of Pseudomonas aeruginosa reveals adaptation toward iron acquisition from hemoglobin. MBio.

[25] Richardson EJ, Limaye B, Inamdar H, Datta A, Manjari KS, Pullinger GD (2011). Genome sequences of Salmonella enterica serovar typhimurium, Choleraesuis, Dublin, and Gallinarum strains of well- defined virulence in food-producing animals. J Bacteriol.

[26] Wallis TS, Paulin SM, Plested JS, Watson PR, Jones PW (1995). The Salmonella dublin virulence plasmid mediates systemic but not enteric phases of salmonellosis in cattle. Infect Immun.

[27] Jelsbak L, Thomsen LE, Wallrodt I, Jensen PR, Olsen JE (2012). Polyamines are required for virulence in Salmonella enterica Serovar Typhimurium. PLoS One.

[28] Wallrodt I, Jelsbak L, Thomsen LE, Brix L, Lemire S, Gautier L (2014). Removal of the phage-shock protein PspB causes reduction of virulence in Salmonella enterica serovar Typhimurium independently of NRAMP1. J Med Microbiol.

[29] Datsenko KA, Wanner BL (2000). One step inactivation of chromosomal genes in Escherichia coli K-12 using PCR products. Proc Natl Acad Sci U S A.

[30] Wallrodt I, Jelsbak L, Thorndahl L, Thomsen LE, Lemire S, Olsen JE (2013). The putative thiosulfate sulfurtransferases PspE and GlpE contribute to virulence of Salmonella Typhimurium in the mouse model of systemic disease. PLoS One.

[31] Jelsbak L, Ingmer H, Valihrach L, Cohn MT, Christiansen MH, Kallipolitis BH (2010). The chaperone ClpX stimulates expression of Staphylococcus aureus protein A by Rot dependent and independent pathways. PLoS One.

[32] Chang AC, Cohen SN (1978). Construction and characterization of amplifiable multicopy DNA cloning vehicles derived from the P15A cryptic miniplasmid. J Bacteriol.

