# Supplementary material for: Salmonella Typhimurium undergoes distinct genetic adaption during chronic infections of mice
Source: BMC Microbiol. 2016 Mar 8;16:30. doi: 10.1186/s12866-016-0646-2 (PMC4784465; doi:10.1186/s12866-016-0646-2)
Supplement: Additional file 1: Table S1. — List of primers used in the study. (DOCX 17 kb) [file 12866_2016_646_MOESM1_ESM.docx]

Additional file 1: Table S1

Primers used in the study

| Primer | Sequence 5´->3´ |
| --- | --- |
| Tag1kanfwd | cgcgcgataggtgtggagtggtgtcgttgggggggttgttTTGTGTCTCAAAATCTCTGATGTTACATTGCAC |
| Tag2kanfwd | ggggagttgttggtattgcgggtggtagctggtgggagctTTGTGTCTCAAAATCTCTGATGTTACATTGCAC |
| Tag3kanfwd | ggcgagggcgcgagtgagatcgagtgtgtgggattgatatTTGTGTCTCAAAATCTCTGATGTTACATTGCAC |
| Tag4kanfwd | agctggagctcgagagtgagtgaggggtgtctttagctatTTGTGTCTCAAAATCTCTGATGTTACATTGCAC |
| Tag5kanfwd | ggcgtgggagtgagggggtgggctggtgagcgttatagttTTGTGTCTCAAAATCTCTGATGTTACATTGCAC |
| Tag6kanfwd | aggggtggggttatggggctggatagtgcgcgtgctagctTTGTGTCTCAAAATCTCTGATGTTACATTGCAC |
| Tag7kanfwd | attgggatggcggtatagttaggtatatcgttcttgatttTTGTGTCTCAAAATCTCTGATGTTACATTGCAC |
| Tag8kanfwd | agagagagctggagagtgatcggtggttgtggtgggagtgTTGTGTCTCAAAATCTCTGATGTTACATTGCAC |
| tagkanrev2 | gctctgccagtgttacaaccaattaacc |
| Tag1malXYfwd | caatcacgcgacaggcatcgtacagctcaatcagcataatttgcgcgcgataggtgtggagtgg |
| Tag2malXYfwd | caatacagcgacaggcatcgtacagctcaatcagcataatttgggggagttgttggtattgcgggtg |
| Tag3malXYfwd | caatcacgcgacaggcatcgtacagctcaatcagcataatttgggcgagggcgcgagtgagat |
| Tag4malXYfwd | caatcacgcgacaggcatcgtacagctcaatcagcataatttgagctggagctcgagagtgagtgag |
| Tag5malXYfwd | caatcacgcgacaggcatcgtacagctcaatcagcataatttgggcgtgggagtgagggggt |
| Tag6malXYfwd | caatcacgcgacaggcatcgtacagctcaatcagcataatttgaggggtggggttatggggctg |
| Tag7malXYfwd | caatcacgcgacaggcatcgtacagctcaatcagcataatttgattgggatggcggtatagttaggtatatcg |
| Tag8malXYfwd | caatcacgcgacaggcatcgtacagctcaatcagcataatttgagagagagctggagagtgatcggtg |
| Tagsmalxyrev2 | caccatgtcccgcgtcggtcaacttcctgtgaaaaatcgaacagctctgccagtgttacaaccaattaacc |
| tagseqfwd | CCGAACGACGCGCAGCCTCTACTTTTGC |
| tagseqrev | AATCGCGGCCTCGAGCAAGACGTTTCCC |
| kdgR-fwd-XhoI | ctctcgagGCCGTATCTCTGCGGGAGG |
| kdgRrev-BamHI | ctggatcCGTCATGCTAATAACGTTGCTTAGAAGGG |
| kdgR-REDfwd | GCAAACGCAGATCTGGATAAGCAGCCTGATTCTGTATCTTgtgtaggctggagctgcttc |
| kdgR-REDrev | GAAGGGATAATCGTTGTAACCCATTTGTTCAGAAATTTTAcatatgaatatcctccttag |
| ptsNfwd-xhoI | ctctcgagAGTTAGCCAGTGCAGGACGGC |
| ptsNrev-BamHI | ctggatccCTGACGATCATCAGTACCATGTACC |
| ptsN-REDfwd: | ATAAATAACGATACGACTCTACAACTGAGCAGTGTACTTAgtgtaggctggagctgcttc |
| ptsN-REDrev | TGCCTCATTCTGCTCACCTTCGGTGTCAGTAATGATTTGAcatatgaatatcctccttag |
| kanrev | CCGCTTCAGTGACAACGTCGAGCACAGC |
| kdgK-nor-fwd | TCGCAGAAAGGCGCTGATGTTCAGCG |
| kdgK-nor-rev | CGTCTTCATCGTCCAGTGTCAGGAAGG |
| maeB-fwd | GAAACTGGGGCTACAGATCAAAGCG |
| maeB-rev | ATACTTTCCACCAACGCCGCATCAC |
| STM474_0020 fwd | ACAGCAAGGACCGTATGGCTATACC |
| STM474_0020 rev | AGTAGCTGCAAAGAGAGCGTGTCAG |
| yicE-fwd | CGACAAGGGTCGATAAGGTATCGAG |
| yicE-rev | GATTAACAGCGCTGGCGTGATTACC |
| STM474_0057-fwd | TTACTGCACAGGTTCAGCAGCTTCG |
| STM474_0057-rev | TATATGGCGCTGGTGCCGTTAATCC |
